# Learning curve analysis of robotic-assisted thoracoscopic lung resection performed by a novice thoracic surgeon

**DOI:** 10.1016/j.xjon.2026.101850

**Published:** 2026-05-02

**Authors:** Taiki Takasugi, Tohru Mawatari

**Affiliations:** Department of Thoracic Surgery, Hakodate Municipal Hospital, Hakodate, Japan

**Keywords:** robotic-assisted thoracic surgery, learning curve, lung cancer, novice surgeon

## Abstract

**Objectives:**

Robot-assisted thoracoscopic surgery has been increasingly adopted for lung resection, and opportunities for novice surgeons to perform this procedure are expanding. However, evidence regarding the early learning curves among novice thoracic surgeons remains limited. We performed this study to evaluate the learning curve and perioperative outcomes of robot-assisted thoracoscopic surgery for anatomical lung resections performed by novice surgeons.

**Methods:**

We reviewed 30 consecutive patients who underwent robot-assisted thoracoscopic anatomical lung resections between April and December 2025. All procedures were performed by a single novice thoracic surgeon with 4 years of experience in thoracic surgery. The primary outcome was console duration. Secondary outcomes included operative time, estimated blood loss, conversion to thoracotomy, postoperative complications, air leaks, chest drain duration, and length of hospital stay. Learning curve analyses were performed using scatter plots, moving averages, and cumulative sum analyses.

**Results:**

The median console time was 122 minutes (range, 50-221 minutes), which decreased with increasing experience. The median operative time was 186 minutes (range, 97-306 minutes) and showed a similar downward trend. Cumulative sum analysis identified a clear point indicating procedural proficiency after approximately 20 cases. The median blood loss was 20 mL, which was consistently low. One planned conversion to thoracotomy (3.3%) was performed because of severe hilar adhesions. No major postoperative complications (Clavien-Dindo grade ≥III) were observed.

**Conclusions:**

Robot-assisted thoracoscopic anatomical lung resection can be safely performed by novice thoracic surgeons using a structured training framework. Procedural proficiency was achieved in approximately 20 cases, with favorable perioperative outcomes.

**Figure:**
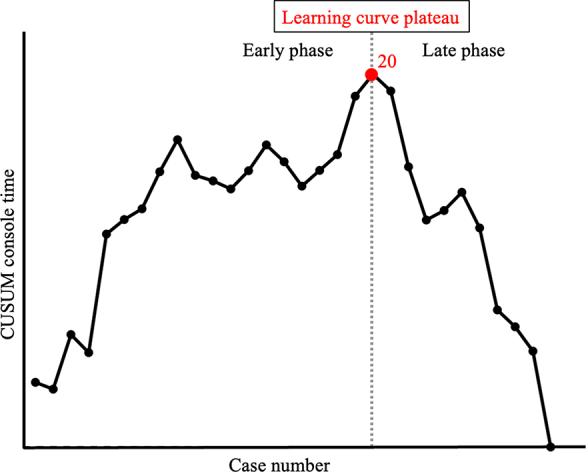
CUSUM analysis identified a learning curve plateau at case 20.

**Central Message**

The CUSUM analysis showed a learning curve plateau in case 20 of RATS performed by a novice surgeon.

**Perspective**

These findings provide practical insights into the early adoption of robot-assisted thoracic surgery by novice surgeons. They may help inform institutional decisions regarding the training structure, case allocation, and supervision strategies to ensure safe implementation.

Robot-assisted thoracoscopic surgery (RATS) is an established minimally invasive approach for anatomical lung resection and is increasingly being incorporated into routine thoracic surgical practice. As its adoption has expanded, the focus of RATS has gradually shifted from feasibility to training and dissemination.^1^

During this transition, surgeons who initially introduced RATS have increasingly assumed supervisory roles, and opportunities for novice thoracic surgeons to perform RATS as primary console surgeons have begun to emerge.^2^ Consequently, RATS is no longer confined to expert operators but is gradually being adopted by the next generation of thoracic surgeons.^3^

The results of previous studies in which investigators evaluated the learning curve of RATS, predominantly involving experienced surgeons, have suggested that procedural proficiency can be achieved in approximately 20 to 30 cases.^4^, ^5^, ^6^, ^7^, ^8^ However, it remains unclear whether these learning curves can be directly applied to novice surgeons at an earlier stage of their surgical careers.

Understanding how novice surgeons acquire procedural proficiency during the early phase of RATS is important to ensure patient safety and to establish appropriate educational and training goals. However, most previous studies evaluating learning curves have focused on experienced surgeons, and data specifically examining the early learning processes of novice surgeons remain limited. The present study characterized the early learning curve of RATS anatomical lung resection performed by a novice thoracic surgeon and examined whether the learning trajectory differed from that previously reported for experienced surgeons.

## Methods

### Study Design and Patients

This study was approved by the institutional review board of Hakodate Municipal Hospital (approval number: 2025-312; approved on January 20, 2026). The requirement for informed consent was waived.

This retrospective observational study included 30 consecutive patients who underwent RATS for anatomical lung resection between April and December 2025. All procedures were performed at a single institution by a single novice thoracic surgeon with 4 years of experience in thoracic surgery at the time of RATS introduction. Patient demographics, operative variables, and postoperative outcomes were collected from the institutional medical records. This study was conducted in accordance with the Declaration of Helsinki, and approval was obtained from the institutional review board.

### Surgeon Background and Training

All procedures were performed by a single novice thoracic surgeon with 4 years of postresidency experience in thoracic surgery at the time of RATS introduction. Before initiating robotic surgery as a primary console surgeon, the surgeon had performed approximately 60 video-assisted thoracoscopic surgery (VATS) anatomical lung resections, including lobectomy and segmentectomy, as the primary operator.

Before the first full-console RATS procedure, the surgeon completed a structured robotic training program that included more than 20 hours of simulation-based training and stepwise console exposure under supervision. During this preparatory phase, the surgeon participated in the robotic procedures with partial console involvement. Specifically, dissection of the upper pulmonary vein and mediastinal lymph nodes at stations #2R and #4R was performed under supervision in 2 separate cases before the surgeon assumed full console responsibility.

Full-console robotic procedures were initiated only after these critical procedural steps were completed and reviewed by surgeons experienced in robotic surgery. Throughout the study period, all the procedures were performed using proctors experienced in robotic surgery who were available for intraoperative supervision to ensure patient safety during the early learning phase.

### Surgical Procedure

RATS for anatomical lung resection was performed with the patient receiving general anesthesia and single-lung ventilation. All procedures were performed using the da Vinci Xi robotic system (Intuitive Surgical) with a 30° endoscopic camera. Carbon dioxide insufflation was used in all cases at a pressure of 8 mm Hg to facilitate exposure of the operative field.

Patients were placed in the lateral decubitus position. A 4-arm robotic approach was used in all procedures. The port placement consisted of three 8-mm robotic ports and one 12-mm robotic port according to a standardized institutional protocol.

A 4-cm utility incision, which primarily served as the assistant port, was created for specimen retrieval using an ALNOTE Wrap Single wound retractor (Alfresa Pharma Corporation). The incision was made at the fourth intercostal space along the anterior axillary line for upper lobe resection and at the fifth intercostal space for middle and lower lobe resection.

None of the patients had chest wall adhesions that were severe enough to preclude port placement. All pleural adhesions were dissected using the robotic console. Lobectomy or anatomical segmentectomy was performed using a standardized anatomy-based approach. Lymph node evaluation was performed in all cases, consisting of either lymph node sampling or systematic mediastinal lymph node dissection, according to the oncologic requirements.

### Statistical Analysis

Continuous variables are summarized as medians with interquartile ranges or as means with ranges, as appropriate. Categorical variables are presented as counts and percentages.

A learning curve analysis was performed using the console time as a measure of procedural proficiency. Temporal changes in the console time were visually assessed using scatter plots and moving averages.

A cumulative sum (CUSUM) analysis was performed to objectively evaluate changes in performance over time.^9^^,^^10^ This method allows the detection of subtle changes in sequential performance that may not be readily captured by conventional descriptive statistics. CUSUM values were calculated as the cumulative sum of the difference between each case's console time and the overall mean console time. An upward trend in the CUSUM curve indicated consecutive cases with longer-than-average console times, whereas a downward trend indicated progressive shortening of the console time. The inflection point of the CUSUM curve was defined as the learning point, which represents the transition from the learning to the proficiency phase. All statistical analyses were performed using the R statistical software (R Foundation for Statistical Computing).

## Results

### Patient Characteristics

Thirty consecutive patients underwent robot-assisted thoracoscopic anatomical lung resection performed by a single novice surgeon during the study period. Patient demographics and baseline characteristics are summarized in Table 1. The median age was 76 years (range, 51-83 years), and 16 patients (53%) were male. The median body mass index was 22.9 kg/m^2^ (range, 17.2-33.8 kg/m^2^). Twenty-two patients (73%) had a history of smoking, with a median smoking exposure of 43 pack-years (range, 0.1-72 pack-years).

**Table 1:** Patient characteristics (n = 30)

Variable	Value
Number of patients	30
Age, y (range)	76 (51-83)
Sex	
Male	16 (53%)
Female	14 (47%)
Body mass index, kg/m^2^ (range)	22.9 (17.2-33.8)
Smoking history	
Ever-smokers	22 (73%)
Smoking exposure, pack-years (range)	43 (0-72)
Diagnosis	
Primary lung cancer	26 (87%)
Metastatic lung tumor	3 (10%)
Benign disease	1 (3%)
Clinical stage (primary lung cancer only, n = 26)	
Stage I	18 (69%)
Stage II-III	8 (31%)
Surgical procedure	
Lobectomy	25 (83%)
Anatomical segmentectomy	5 (17%)
Type of resection	
Right upper lobectomy	8 (26.7%)
Right middle lobectomy	7 (23.3%)
Right lower lobectomy	6 (20.0%)
Left upper lobectomy	3 (10.0%)
Left lower lobectomy	1 (3.3%)
Segmentectomy (S6, LS1 + 2, left upper division)	5 (16.7%)
Side of surgery	
Right	23 (77%)
Left	7 (23%)
Lymph node evaluation	
Sampling	13 (43%)
Systematic mediastinal dissection	17 (57%)

Data are presented as median (range) or number (%), unless otherwise indicated.

Primary lung cancer accounted for 26 cases (87%), whereas 3 patients (10%) had metastatic lung tumors and 1 patient (3%) had benign disease. Among patients with primary lung cancer, 18 (69%) were classified as clinical stage I and 8 (31%) as stages II to III.

Regarding surgical procedures, lobectomy was performed in 25 patients (83%) and anatomical segmentectomy in 5 patients (17%). The right lung was operated on in 23 patients (77%), and the left lung was operated on in 7 patients (23%). Lymph node evaluation included lymph node sampling in 13 patients (43%) and systematic mediastinal lymph node dissection in 17 patients (57%).

### Perioperative Outcomes

Perioperative outcomes are summarized in Table 2. The median console time was 122 minutes (range, 50-221 minutes), and the median operative time was 186 minutes (range, 97-306 minutes). The median estimated blood loss was 20 mL (range, 5-70 mL).

**Table 2:** Perioperative outcomes (n = 30)

Outcome	Value
Number of patients	30
Operative time, min	186 (97-306)
Console time, min	122 (50-221)
Estimated blood loss, mL	20 (5-70)
Planned conversion to thoracotomy, n (%)	1 (3.3%)
Air leak, n (%)	2 (6.7%)
Chest tube duration, d	2 (1-7)
Postoperative length of hospital stay, d	5 (3-22)
Postoperative complications (Clavien-Dindo ≥II), n (%)	2 (6.7%)
Reoperation within 30 d, n (%)	0 (0%)
30-d mortality, n (%)	0 (0%)

Shown are the perioperative outcomes of patients who underwent robotic anatomical lung resection. Data are presented as median (range) or number (%), unless otherwise indicated.

One patient (3.3%) underwent a planned conversion to thoracotomy because of severe hilar adhesions involving the pulmonary artery and superior vena cava. Air leaks occurred in 2 patients (6.7%). The median chest tube duration was 2 days (range, 1-7 days). The median postoperative length of hospital stay was 5 days (range, 3-22 days). Postoperative complications of Clavien-Dindo grade II occurred in 2 patients (6.7%), including 1 case of atrial fibrillation and 1 case of acute exacerbation of interstitial pneumonia, both of which were treated with medical therapy. None of the patients experienced complications of Clavien-Dindo grade III or greater.

### Learning Curve Analysis

**Figure 1:**
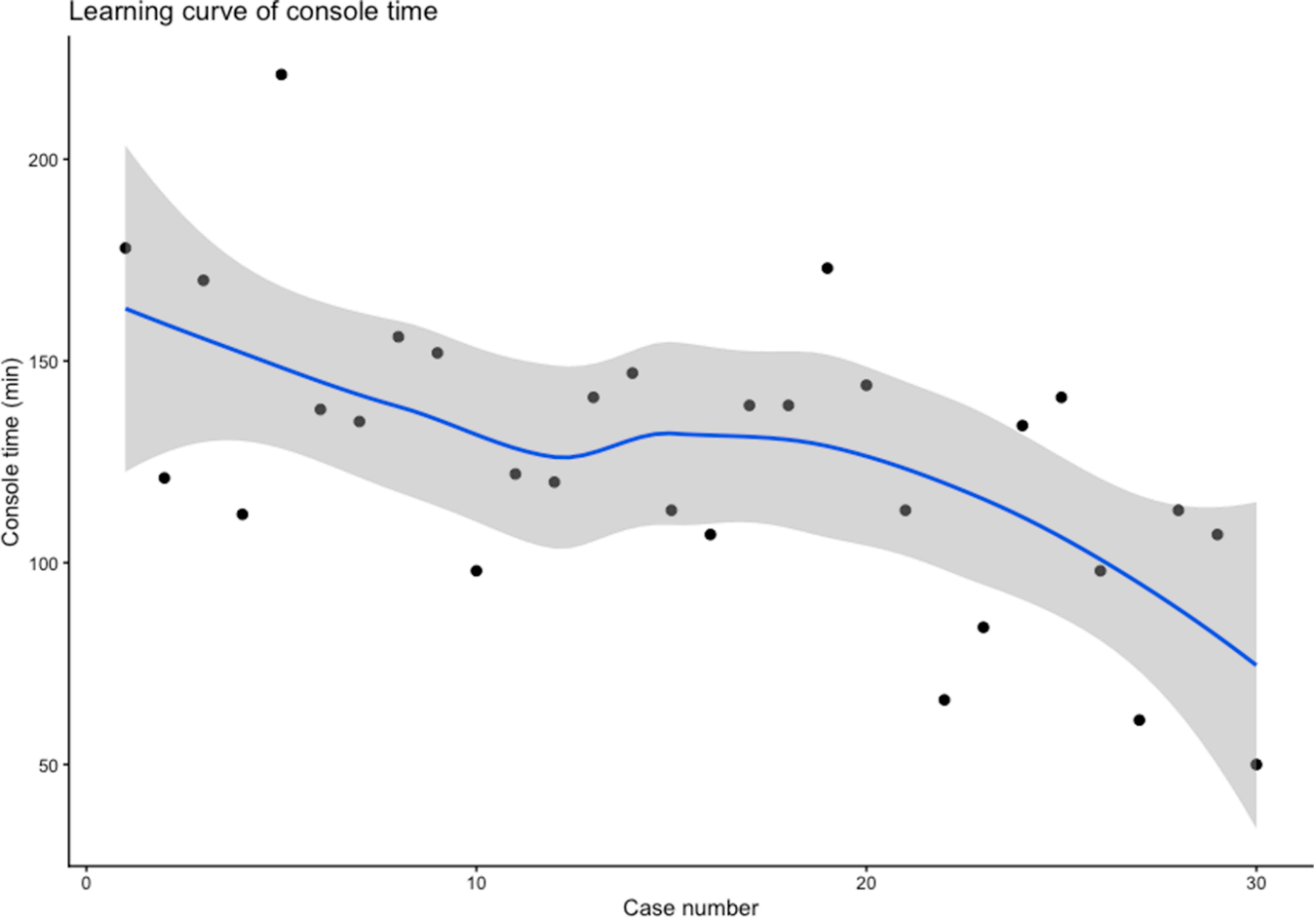
Learning curve of console time. The scatter plot depicts the console time according to the case sequence. The LOESS (ie, LOcally WEighted Scatter-plot Smoother) curve (*blue line*) demonstrates a progressive decrease in console time, with a plateau phase emerging at case 20, which is consistent with the learning curve plateau identified by the CUSUM analysis. *CUSUM*, Cumulative sum.

**Figure 2:**
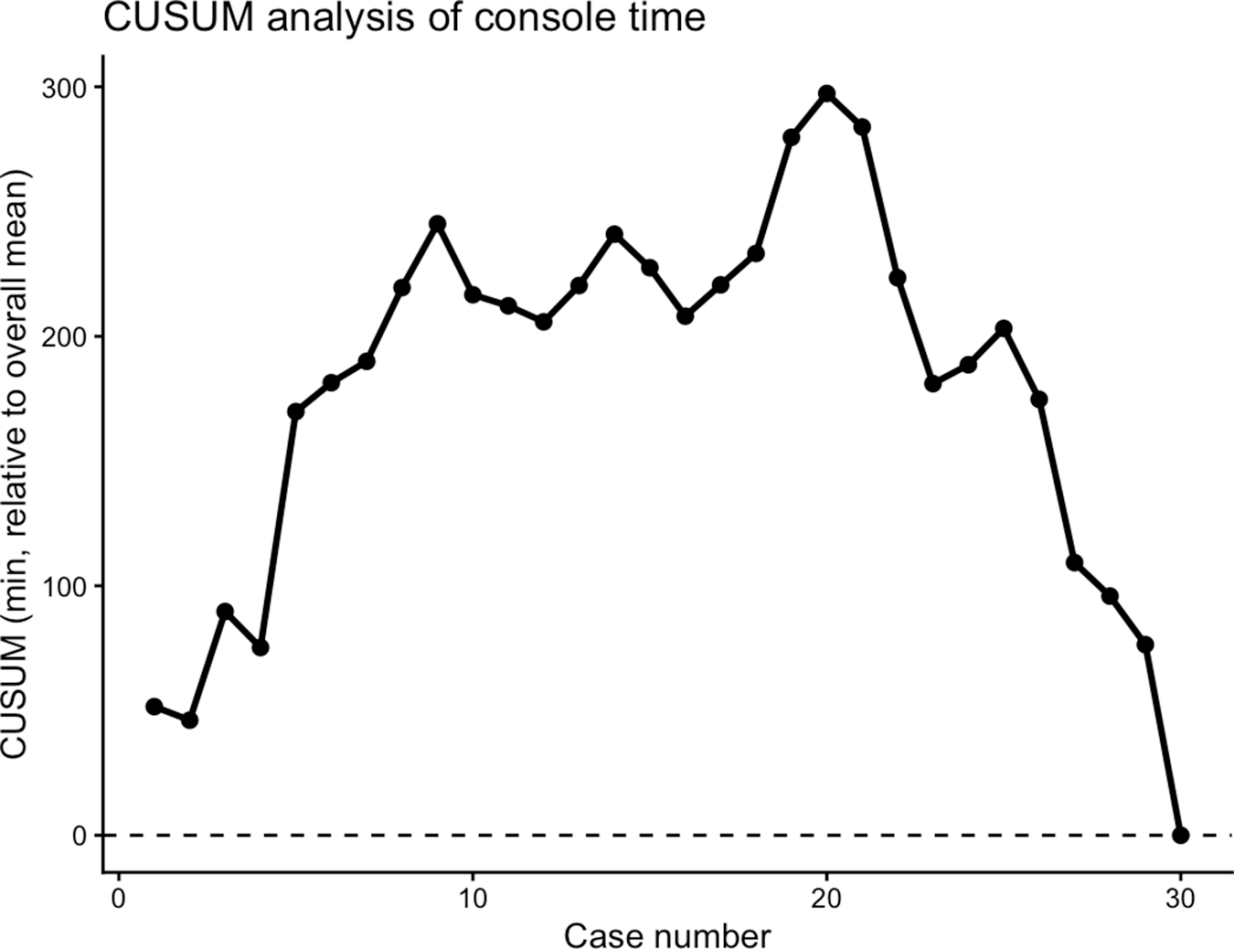
Cumulative sum (*CUSUM*) analysis of console time. The CUSUM values were calculated as the cumulative sum of the difference between each case's console time and the overall mean console time. The CUSUM curve increased during the early phase, peaked at case 20, and subsequently demonstrated a downward trend, indicating a transition from the learning phase to the more proficient phase.

The learning curve of console time is shown in Figure 1, which demonstrates a gradual reduction in console time as surgical experience accumulates. To further evaluate the learning process, a CUSUM analysis of the console time was performed, as shown in Figure 2.

CUSUM analysis revealed an initial upward trend followed by a sustained downward slope, indicating a progressive improvement in operative efficiency. The turning point of the learning curve was observed at case 20, which was considered to be the learning curve plateau.

### Comparison Between Early and Late Phases

On the basis of the CUSUM-defined learning curve plateau in case 20, patients were divided into early phase (cases 1-20) and late phase (cases 21-30). A comparison of perioperative outcomes between the 2 phases is summarized in Table 3. No significant differences were found between the 2 phases in terms of the surgical procedure, clinical stage of primary lung cancer, or smoking history.

**Table 3:** An exploratory comparison before and after the learning curve plateau

Outcome	Early phase (cases 1-20)	Late phase (cases 21-30)	*P* value
Number of patients	20	10	–
Operative time, min	203 (141-306)	146 (97-186)	**.0023**
Console time, min	139 (98-221)	102.5 (50-141)	**.0015**
Estimated blood loss, mL	20 (5-50)	15 (5-70)	.437
Conversion to thoracotomy, n (%)	1 (5%)	0 (0%)	1
Postoperative complications (Clavien-Dindo ≥II), n (%)	2 (10%)	0 (0%)	.54
Reoperation within 30 d, n (%)	0 (0%)	0 (0%)	–
30-d mortality, n (%)	0 (0%)	0 (0%)	–
Chest tube duration, d	2 (2-7)	2 (1-2)	**.0117**
Surgical procedure, n (%)			
Lobectomy	16 (80%)	9 (90%)	.64
Segmentectomy	4 (20%)	1 (10%)	
Clinical stage (primary lung cancer)	n = 17	n = 8	
Stage I	12 (71%)	6 (75%)	1
Stage II-III	5 (29%)	2 (25%)	
Ever-smoker, n (%)	15 (75%)	7 (70%)	1

Patients were divided into early phase (cases 1-20) and late phase (cases 21-30) on the basis of the CUSUM-defined learning curve plateau. Data are presented as median (range) or number (%) unless otherwise indicated. Bold values indicate statistical significance. *CUSUM*, Cumulative sum.

The median operative time was significantly shorter in the late phase than in the early phase (203 minutes [range, 141-306 minutes] vs 146 minutes [range, 97-186 minutes], respectively; *P* = .0023). Similarly, median console time was significantly reduced after the learning curve plateau (139 minutes [range, 98-221 minutes] vs 102.5 minutes [range, 50-141 minutes], *P* = .0015).

No significant differences were observed in estimated blood loss between the early and late phases (20 mL [range, 5-50 mL] vs 15 mL [range, 5-70 mL], *P* = .437). Postoperative complications of Clavien-Dindo grade II or greater occurred in 2 patients (10%) in the early phase and none in the late phase, without a statistically significant difference (*P* = .54). One patient in the early phase underwent a planned conversion to thoracotomy, whereas no conversion occurred in the late phase (*P* = 1.00).

Chest tube duration was significantly shorter in the late phase than in the early phase (2 days [range, 2-7 days] vs 2 days [range, 1-2 days]; *P* = .0117). None of the patients required reoperation within 30 days, and no 30-day mortality was observed during either phase.

## Discussion

RATS has been rapidly adopted in recent years and is no longer limited to highly specialized academic centers but is now increasingly feasible in community hospitals.^11^ To the best of our knowledge, RATS has been predominantly introduced and performed by surgeons with extensive experience in VATS, and a substantial body of literature has accumulated regarding the learning curves and perioperative outcomes in this context.^4^, ^5^, ^6^, ^7^, ^8^ In contrast, reports focusing specifically on the learning processes of novice surgeons who act as the primary console operators remain limited.

Recent studies have suggested that the learning curve for RATS may be shorter than that for VATS because of the advantages of instrument articulation, visualization, and ergonomics.^12^, ^13^, ^14^ These features have attracted attention to RATS as a minimally invasive approach that can be adopted more readily by novice surgeons.^15^ Against this background, RATS is gradually being disseminated not only among experienced surgeons but also among novice surgeons, indicating that thoracic surgery is currently in a transitional phase toward robotic surgery.

In the present study we evaluated the learning curve of robot-assisted thoracoscopic anatomical lung resection performed by a novice thoracic surgeon with 4 years of experience in thoracic surgery. Using CUSUM analysis of the console time, we identified a learning curve plateau at 20 cases. Importantly, operative efficiency improved significantly beyond this point without compromising perioperative safety, suggesting that stepwise skill acquisition is achievable even for novice surgeons under an appropriate educational framework (Video 1).

Previous studies evaluating the learning curve of RATS lobectomy have reported that proficiency is generally achieved in approximately 20 to 30 cases.^4^, ^5^, ^6^, ^7^, ^8^^,^^14^^,^^16^^,^^17^ Specifically, Arnold and colleagues^5^ reported a learning curve plateau at 22 cases, Paglialunga and colleagues at 23 cases,^6^ and Toker and colleagues^18^ at 14 cases. Furthermore, a meta-analysis integrating multiple studies estimated that 25.3 ± 12.6 cases are required to achieve proficiency. Consistent with this finding, another meta-analysis reported a median of 20 cases with an interquartile range of approximately 13-27, indicating a comparable range for proficiency attainment.^8^ Similarly, studies focusing on robotic segmentectomy have reported learning curve plateaus in approximately 20 to 30 cases; Igai and colleagues^19^ reported 22 cases, and Le Gac and colleagues^20^ reported 27 cases. Notably, these reports uniformly analyzed a single surgeon with substantial previous experience with VATS.

Although the present analysis also involved a single surgeon, the operator differed substantially from previous reports in that the surgeon was in the early stages of thoracic surgical training. Despite this difference, the learning curve observed in the present study plateaued after approximately 20 cases, which is comparable with that reported for surgeons who are deemed proficient in VATS. The similarity in the learning curve plateaus between the present study and previous reports may be explained by several factors. First, the surgical approach in RATS was intentionally designed to closely mirror the approach previously used during VATS, helping to maintain procedural familiarity. Second, operative steps were consistently reviewed and confirmed by a supervising surgeon before each procedure, contributing to standardized decision-making and intraoperative strategies. Third, surgical devices commonly used in VATS, such as Maryland-type instruments, have also been used in RATS to minimize the learning burden associated with unfamiliar instrumentation. Furthermore, RATS itself may reduce interindividual variability as the result of its intrinsic advantages such as tremor filtration, 3-dimensional visualization, and surgeon-controlled camera manipulation.^21^ Collectively, these factors may mitigate differences related to surgeon seniority and contribute to a learning curve comparable with that reported in previous studies. To the best of our knowledge, this study provides early evidence that novice thoracic surgeons can achieve a learning curve comparable with that of experienced VATS surgeons for RATS anatomical lung resection.

In addition, the relatively high case acquisition rate in this study may have contributed to more continuous skill acquisition and earlier performance stabilization. Because case volume is highly dependent on institutional factors, the findings should be interpreted with caution when applied to centers with lower procedural volumes.

Furthermore, whereas most previous studies focused exclusively on lobectomy, the present study included anatomical lung resections comprising both lobectomy and segmentectomy. Segmentectomy is generally considered more technically demanding than lobectomy because of the need for precise intersegmental anatomical understanding and parenchymal division.^22^^,^^23^ Despite this heterogeneity in surgical procedures, the learning curve observed in the present study remained comparable with that reported for lobectomy-only cohorts. This finding strongly suggests that with appropriate case selection and structured training, novice surgeons may achieve early proficiency even in technically complex robotic anatomical lung resections.

Perioperative safety was well maintained throughout the study period, including during the early learning phase. The median console time was 122 minutes (range, 50-221 minutes), which decreased significantly as experience accumulated (*P* = .0023). The median estimated blood loss was consistently low at 20 mL (range, 5-70 mL) regardless of the learning phase. Moreover, the median chest tube duration was 2 days (range, 1-7 days), indicating a favorable postoperative recovery. The median length of hospital stay was 5 days (range, 3-22 days), which is comparable with previously reported outcomes following minimally invasive lung resection.^7^^,^^24^

Only one conversion to thoracotomy (3.3%) occurred, which was a planned conversion attributable to severe hilar adhesions. When introducing RATS to novice surgeons, lowering the threshold for conversion to thoracotomy may play an important role in maintaining perioperative safety.^25^ In particular, in cases with dense lymph node adhesion to the pulmonary artery, as observed in the present case, early and planned conversion should be regarded as an appropriate safety-oriented decision rather than the failure of robotic surgery.

Previous studies have reported postoperative complication rates of approximately 10 to 20% after lung resection.^26^ Postoperative complications were evaluated according to the Clavien-Dindo classification.^27^ In the present study, no Clavien-Dindo grade III or greater postoperative complications were observed, and no increase in complication rates was noted, even during the early learning phase. These findings indicate that even when RATS anatomical lung resection is performed by a novice surgeon as the primary operator, perioperative outcomes comparable with those reported in the literature can be achieved through appropriate case selection, structured training, and flexible surgical strategies, including planned conversion when necessary.

These findings have several important educational implications for training novice thoracic surgeons in robotic surgery. First, sufficient experience with VATS anatomical lung resection before robotic adoption appears to be an important foundation for understanding the hilar anatomy and performing safe vascular dissection.^24^ Although the surgeon's previous experience with VATS was relatively limited compared with that reported in previous studies, the learning curve for VATS anatomical lung resection has been reported to plateau after approximately 35 to 40 cases.^7^^,^^28^^,^^29^ Therefore, the experience of approximately 60 VATS anatomical resections in the present study likely represents a level beyond the initial learning phase, providing an adequate foundation for the safe introduction of RATS.

Moreover, preoperative proctoring, team-based discussion of surgical approaches, and clear role allocation between the surgeon and assistant are essential to ensure safety during the early robotic experience. In this study, the surgical procedures and key technical points were thoroughly reviewed preoperatively, and team-based preparations may have contributed to the favorable perioperative outcomes observed.

Furthermore, stepwise training, in which key procedural components are mastered before assuming full responsibility for the console, appears to be an effective educational strategy.^30^ Cerfolio and colleagues^31^ reported that the standardization of surgical workflows into predefined procedural steps is beneficial for training novice surgeons in RATS. Similarly, in the present study, a standardized surgical approach may have contributed to stabilization of the learning curve.

Finally, novice surgeons are often required to perform RATS and VATS in routine clinical practice. Experience with robotic surgery, including 3-dimensional visualization and high-definition imaging, may enhance the anatomical recognition and technical precision of thoracoscopic surgery.^26^ Thus, experience with RATS may confer synergistic benefits, contributing not only to proficiency in robotic surgery itself but also to the overall improvement in minimally invasive thoracic surgical techniques.

The present study has several limitations. First, this retrospective analysis was conducted at a single institution and involved a single surgeon, which may have limited the generalizability of the findings. The backgrounds and training environments of surgeons vary across institutions, and the results may not be directly applicable to all novice surgeons.

Second, the sample size was relatively small (30 patients), and long-term oncologic outcomes could not be evaluated. However, the number of cases analyzed fell within the range reported in previous meta-analyses of robotic learning curves.^4^ Because the shape and peak of the CUSUM curve may depend on the number of cases analyzed, the findings should be interpreted in this context. Moreover, the inclusion of both lobectomies and segmentectomies introduced variability in surgical complexity, including differences in fissure development and adhesion, which may have influenced the learning curve. Finally, the console time was used as the primary metric for learning curve assessment; however, it was difficult to fully adjust for factors such as case complexity and anatomical variations.

## Conclusions

This study evaluated the learning curve and perioperative outcomes of robot-assisted thoracoscopic anatomical lung resection performed by a novice thoracic surgeon with 4 years of surgical experience. The learning curve, assessed using console time, reached a plateau in at 20 cases, which was comparable with previously reported learning curves achieved by surgeons who are experienced in VATS. Importantly, perioperative safety was maintained throughout the learning period, including the early adoption phase.

These findings suggest that RATS for anatomical lung resection can be safely and efficiently performed by novice thoracic surgeons within an appropriate educational framework. These results support the feasibility of the early adoption of RATS by novice surgeons and provide practical insights for robotic surgery training and dissemination in thoracic surgery.

### Declaration of Generative AI and AI-Assisted Technologies in the Writing Process

During the preparation of this work, the authors used ChatGPT (OpenAI) in order to assist with language editing and refinement of the manuscript. After using this tool/service, the authors reviewed and edited the content as needed and take full responsibility for the content of the publication.

## Conflict of Interest Statement

The authors reported no conflicts of interest.

The *Journal* policy requires editors and reviewers to disclose conflicts of interest and to decline handling or reviewing manuscripts for which they may have a conflict of interest. The editors and reviewers of this article have no conflicts of interest.

## Supplementary Data

Video 1 Standardized robot-assisted thoracoscopic right upper lobectomy after completing the learning curve. The video shows representative operative steps, including port placement, hilar dissection, fissure management, and lymph node dissection. Video available at: https://www.jtcvs.org/article/S2666-2736(26)00273-1/fulltext.
